# Effects of Tirzepatide on Patients With Type 2 Diabetes and Metabolic Dysfunction-Associated Steatotic Liver Disease: A Retrospective Cohort Study

**DOI:** 10.7759/cureus.83712

**Published:** 2025-05-08

**Authors:** Hideyuki Okuma

**Affiliations:** 1 Department of Diabetes and Endocrinology, Graduate School of Interdisciplinary Research, Faculty of Medicine, University of Yamanashi, Yamanashi, JPN

**Keywords:** body weight loss, glucagon-like peptide-1 agonist (glp-1ra), masld, tirzepatide, type 2 diabetes

## Abstract

Background: Tirzepatide, a glucose-dependent insulinotropic polypeptide/glucagon-like peptide-1 (GLP-1) receptor agonist, has recently been introduced in Japan; however, there are limited studies on its effectiveness in Japanese patients with type 2 diabetes diagnosed with metabolic dysfunction-associated steatotic liver disease (MASLD).

Patients and methods: This retrospective cohort study was conducted among 54 Japanese patients (29 men and 25 women) with type 2 diabetes and MASLD to assess the impact of switching from a GLP-1 receptor agonist (GLP-1RA) to tirzepatide. Before the switch, either dulaglutide or semaglutide was used as the GLP-1RA. Clinical findings were analyzed before and six months after switching to tirzepatide. Additionally, a multiple regression analysis was performed to determine whether characteristics and test results before switching to tirzepatide could predict the weight loss and MASLD suppression six months after initiation. The fatty liver index and fibrosis-4 (FIB-4) index were utilized as MASLD indicators. High-sensitivity C-reactive protein (hsCRP) levels from residual serum were measured as an indicator of chronic inflammation.

Results: Six months after switching to tirzepatide, significant reductions in body weight, hemoglobin A1c (HbA1c) level, fatty liver index, FIB-4 index, and hsCRP level were observed. The multiple regression analysis identified age, duration of type 2 diabetes, and HbA1c levels before the switch as significant independent predictors of weight loss rate. Also, the multiple regression analysis suggested that age before the switch may serve as a useful predictor of a decrease in fatty liver index. The effect of tirzepatide on appetite was less pronounced in the group that had used semaglutide before the switch compared with the group that had used dulaglutide; however, even in the semaglutide group, significant reductions in body weight, HbA1c levels, fatty liver index, and FIB-4 index were noted six months after the switch.

Conclusions: This study suggested the efficacy of switching from GLP-1RAs to tirzepatide among Japanese patients with type 2 diabetes and MASLD. Predictors of weight loss and fatty liver index reduction after switching to tirzepatide were identified. Additionally, we found that the therapeutic effect of tirzepatide can be expected even in patients who were using semaglutide before the switch.

## Introduction

Metabolic dysfunction-associated steatotic liver disease (MASLD), a form of fatty liver disease predominantly linked to metabolic syndrome, is rapidly increasing worldwide [1-3]. As MASLD advances from simple fatty liver disease to metabolic dysfunction-associated steatohepatitis (MASH), it can progress to liver cirrhosis and hepatocellular carcinoma [3-6]. The rise in obesity and type 2 diabetes has contributed to the growing prevalence of MASH [7], which is associated with both cardiovascular disease and liver-related mortality [4,8]. Consequently, various therapeutic agents are being developed to address this condition.

Recently, the connection between type 2 diabetes and MASLD has gained significant attention, with evidence confirming that diabetes medications, including thiazolidinediones, glucagon-like peptide-1 receptor agonists (GLP-1RAs), and sodium-glucose cotransporter 2 inhibitors (SGLT2is), are effective in treating fatty liver [9-11]. Emerging evidence also suggests that SGLT2is can improve MASH and liver fibrosis [12-14]. In contrast, GLP-1RAs are effective in treating MASH but not in improving liver fibrosis [15]. Glucose-dependent insulinotropic polypeptide (GIP)/GLP-1RA tirzepatide [16,17] has been shown to significantly reduce body weight in placebo-controlled studies involving patients with type 2 diabetes and obesity [16-19], with findings indicating improvement in MASH and liver fibrosis biomarkers in this population [20,21]. Therefore, switching from GLP-1RAs to tirzepatide may benefit patients with inadequate MASLD management despite using GLP-1RAs.

Since tirzepatide was recently introduced in Japan, there are limited studies on its effectiveness in treating fatty liver, MASH, and liver fibrosis in Japanese patients with MASLD after switching from GLP-1RAs to tirzepatide. Therefore, this study aimed to confirm whether switching to tirzepatide has a positive effect on MASLD in Japanese patients with type 2 diabetes and MASLD. In this study, we performed a retrospective analysis of the therapeutic effects of switching from GLP-1RAs to tirzepatide in patients with type 2 diabetes and MASLD. As a result, six months after switching to tirzepatide, reductions in body weight and HbA1c were observed, along with decreases in the liver disease markers fatty liver index and fibrosis-4 (FIB-4) index. The amount of weight loss was significantly correlated with the reduction in the fatty liver index, suggesting that tirzepatide's improvement in MASLD is dependent on weight loss. Tirzepatide is expected to be a useful medication for patients with MASLD, a social issue, particularly those with obesity.

## Materials and methods

Participants and survey period

**Figure 1:**
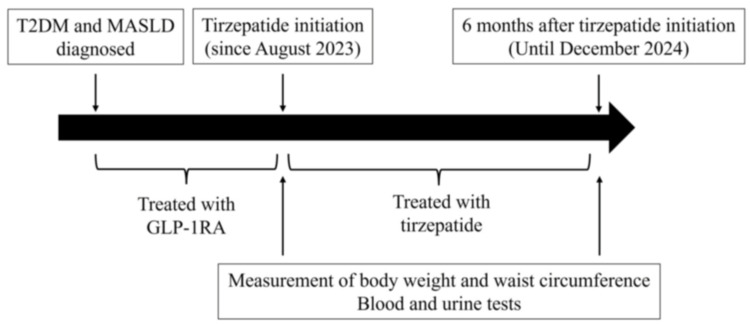
Time course of this study T2DM, type 2 diabetes mellitus; MASLD, metabolic dysfunction associated steatotic liver disease; GLP-1RA, glucagon-like peptide-1 receptor agonist

We enrolled Japanese patients with type 2 diabetes from the internal medicine outpatient clinic at Nerima Hikarigaoka Hospital who had confirmed fatty liver disease through imaging tests (abdominal ultrasound, computed tomography, and magnetic resonance imaging). These patients had been treated with GLP-1RAs either alone or in combination with oral hypoglycemic agents or insulin for more than six months. They switched from GLP-1RAs to tirzepatide 2.5 mg/week after August 2023, with the dose increased to 5 mg/week four weeks later, and they used tirzepatide for a total of six months or more as of December 2024. The therapeutic effects of tirzepatide over the six-month period were then examined in this patient group (Figure 1).

**Figure 2:**
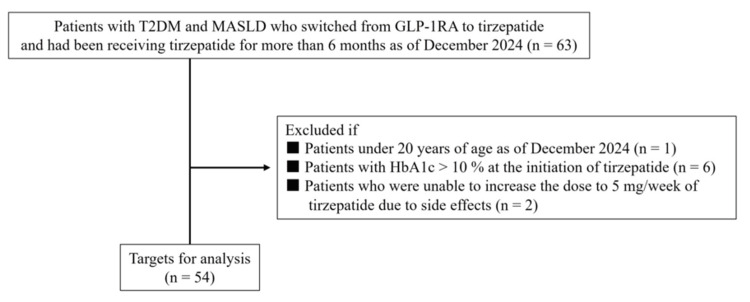
Flowchart of patient selection in this study After nine patients were excluded from a total of 63 Japanese patients with T2DM and MASLD according to the set exclusion criteria, the remaining 54 patients were included in the analysis T2DM, type 2 diabetes mellitus; MASLD, metabolic dysfunction-associated steatotic liver disease; GLP-1RA, glucagon-like peptide-1 receptor agonist; HbA1c: hemoglobin A1c

Imaging tests were conducted within six months before switching to tirzepatide for all patients for diagnosing the presence of fatty liver [3]. The current study included 54 patients, excluding one under 20 years of age as of December 2024, six with a hemoglobin A1c (HbA1c) level of 10% or higher before switching to tirzepatide, and two who could not increase the tirzepatide dose to 5 mg/week due to side effects such as nausea and abdominal pain (Figure 2).

Among patients with HbA1c >10%, many require insulin to relieve hyperglycemia, and there is a possibility that cases of “diabetic precoma,” which is a contraindication for tirzepatide, may be included. Therefore, these patients were excluded from the target population. The study period spanned from July 2023 to January 2025, with medical records from daily care retrospectively analyzed. The study protocol was approved by the certified review board of Nerima Hikarigaoka Hospital.

Survey items

We surveyed age, sex, body weight, body mass index (BMI), waist circumference, duration of type 2 diabetes, HbA1c, aspartate aminotransferase (AST), alanine aminotransferase (ALT), gamma-glutamyl transpeptidase (γ-GTP), triglycerides (TGs), non-high-density lipoprotein cholesterol (non-HDL-C), estimated glomerular filtration rate (eGFR), urine albumin-to-creatinine ratio (UACR), high-sensitivity C-reactive protein (hsCRP), fatty liver index, FIB-4 index, and the use of SGLT2is, metformin, insulin, statins, or fibrates. Additionally, the type of GLP-1RA used before switching to tirzepatide and whether appetite suppression occurred due to tirzepatide were noted. The UACR was measured through random urine tests at the outpatient clinic and converted to grams of creatinine (measured in mg/g). Additionally, residual blood samples before switching to tirzepatide were used to measure serum hsCRP levels. Serum hsCRP levels were measured using the immunoturbidimetric CRP-Latex assay (Kamiya Biomedical Co., Tukwila, WA), following the manufacturer’s protocol. The fatty liver index, developed by Bedogni et al., is a measure of fatty liver disease calculated using a formula that incorporates waist circumference, BMI, and TG and γ-GTP levels. A value between 30 and 59 suggests the presence of fatty liver, while a value of 60 or higher strongly indicates fatty liver [22]. This index is also commonly used to assess the progression of MASLD [23,24]. The FIB-4 index is a scoring system used to evaluate the degree of liver fibrosis based on AST, ALT, platelet count, and age [25]. A score ranging from 1.3 to 2.67 suggests the possibility of fibrosis progression, while a score of 2.67 or higher indicates that more than half of the patients may have liver cirrhosis or are close to developing it [26]. This index is widely utilized as a marker of disease progression in MASH [27]. The presence or absence of appetite suppression due to tirzepatide use was obtained from the electronic medical records recorded by the attending physician during the outpatient visit six months after switching to tirzepatide.

Evaluatory items

To evaluate efficacy, we conducted an analysis of changes in clinical findings before and six months after switching to tirzepatide, which included body weight, BMI, waist circumference, HbA1c, AST, ALT, γ-GTP, TGs, non-HDL-C, eGFR, UACR, hsCRP, the fatty liver index, and the FIB-4 index. We also performed a correlation analysis between changes in each parameter and changes in the fatty liver index, FIB-4 index, and UACR. Additionally, a stratified analysis was conducted based on the presence or absence of appetite suppression due to tirzepatide, the use of SGLT2is, and the type of GLP-1RAs used before switching to tirzepatide. Furthermore, simple and multiple regression analyses were performed using the weight loss rate and the decrease in fatty liver index six months after switching to tirzepatide as outcome variables. The change (Δ) in each parameter was calculated by subtracting the value before switching to tirzepatide from the value six months after the switch. The weight loss rate was calculated by subtracting the weight six months after switching to tirzepatide from the weight before the switch, dividing the result by the weight before the switch, and multiplying by 100. The reduction in the fatty liver index was determined by subtracting the fatty liver index six months after switching to tirzepatide from the fatty liver index before the switch.

Statistical analysis

Values are expressed as means ± standard deviations for normally distributed data and medians (ranges) for nonnormally distributed data. A paired t-test or Wilcoxon signed-rank sum test was used for time-course evaluation against preswitch values. Pearson's correlation coefficient was applied for correlation analysis, with nonnormally distributed values logarithmically transformed before analysis. The Student's t-test, Mann-Whitney U test, and Fisher's exact test were used for intergroup comparisons. Logistic regression analysis was used for comparative analysis, adjusted for age. All statistical analyses were performed using Prism 7 software (GraphPad Software Inc., San Diego, CA), with statistical significance set at p < 0.05.

## Results

Patient background

The clinical characteristics of the enrolled subjects before switching to tirzepatide are summarized in Table 1.

**Table 1:** Clinical characteristics of the enrolled subjects before switching to tirzepatide Data are expressed as the mean ± standard deviation, the median (range), or number (%) T2DM, type 2 diabetes mellitus; HbA1c, hemoglobin A1c; AST, aspartate aminotransferase; ALT, alanine aminotransferase; γ-GTP, γ-glutamyl transpeptidase; TG, triglyceride; non-HDL-C, non-high-density lipoprotein cholesterol; eGFR, estimated glomerular filtration rate; UACR, urine albumin-to-creatinine ratio; hsCRP, high sensitive C-reactive protein; FIB-4, fibrosis 4; SGLT2i, SGLT2, sodium glucose co-transporter 2 inhibitor; GLP-1RA, glucagon-like peptide-1 receptor agonist

Parameters	All subjects (n = 54)
Females, n (%)	25 (46)
Age (years)	57.2 ± 10.5
Body weight (kg)	71 (53.2-123.8)
Body mass index (kg/m^2^)	26.8 (23-43.8)
Waist circumference (cm)	88 (74-112)
Duration of T2DM (years)	7.5 (1-29)
HbA1c (%)	8.17 ± 0.97
AST (U/L)	29 (11-173)
ALT (U/L)	39 (13-169)
γ-GTP (U/L)	54 (13-163)
TG (mg/dL)	246 (77-619)
non-HDL-C (mg/dL)	140.5 (115-165)
eGFR (mL/minute/1.73 m^2^)	71.5 (54-85)
UACR (mg/g)	35 (3-112)
hsCRP (mg/L)	3 ± 0.85
Fatty liver index	66.8 ± 16.9
FIB-4 index	1.30 ± 0.54
SGLT2i, n (%)	33 (61)
GLP-1RA before tirzepatide initiation, n (%)	
Dulaglutide	24 (44)
Semaglutide	30 (56)
Metformin	47 (87)
Insulin	8 (15)
Statin	32 (59)
Fibrate	35 (65)

The median BMI was 26.8 kg/m² (range: 23-43.8 kg/m²), and the waist circumference was 88 cm (range: 74-112 cm). We did not use tirzepatide in patients with a BMI of less than 23 kg/m^2^, as the electronic package insert for tirzepatide indicated that its efficacy and safety have not been fully studied [28]. The HbA1c level was 8.17% ± 0.97%, exceeding the target range for glycemic control set by the Japan Diabetes Society (HbA1c < 7%). The median liver enzyme levels were elevated, with a predominance of increased ALT levels. The median eGFR was over 60 mL/minute/1.73 m², but the median UACR was 35 mg/g, indicating a high UACR in this group. The hsCRP level was elevated at 3 ± 0.85 mg/L (cutoff > 2), and the fatty liver index was high at 66.8 ± 16.9 (cutoff > 60). The FIB-4 index was 1.30 ± 0.54, with the mean value approaching the threshold (1.3), indicating potential liver fibrosis progression. SGLT2is were concomitantly used by over half of the patients (33 cases, 61%) and continued throughout the six-month study period. Before switching to tirzepatide, all patients were using either dulaglutide (24 cases, 44%) or semaglutide (30 cases, 56%) as their GLP-1RA, with no usage of other GLP-1RA options such as liraglutide. The dosages were 0.75 mg/week for dulaglutide and 1 mg/week for semaglutide. Metformin was concomitantly used in 47 cases (87%), insulin in eight cases (15%), statins in 32 cases (59%), and fibrates in 35 cases (65%), with all these medications maintained throughout the six-month study period.

Changes in parameters before and after switching to tirzepatide

Table 2 presents the changes in each parameter before and six months after switching to tirzepatide.

**Table 2:** Changes in parameters before and after switching to tirzepatide Data are expressed as the mean ± standard deviation or the median (range), and are compared using paired t test, or the Wilcoxon signed-rank sum test HbA1c, hemoglobin A1c; AST, aspartate aminotransferase; ALT, alanine aminotransferase; γ-GTP, γ-glutamyl transpeptidase; TG, triglyceride; non-HDL-C, non-high-density lipoprotein cholesterol; eGFR, estimated glomerular filtration rate; UACR, urine albumin-to-creatinine ratio; hsCRP, high-sensitive C-reactive protein; FIB-4, fibrosis 4

Parameters	All subjects (n = 54)		p value
	Before the switch	Six months after the switch	
Body weight (kg)	71 (53.2-123.8)	67 (54.5-114.7)	<0.0001
Body mass index (kg/m^2^)	26.8 (23-43.8)	26 (22-41.9)	<0.0001
Waist circumference (cm)	88 (74-112)	84 (72-100)	<0.0001
HbA1c (%)	8.17 ± 0.97	7.28 ± 1.34	<0.0001
AST (U/L)	29 (11-173)	25 (10-42)	0.0002
ALT (U/L)	39 (13-169)	30 (12-57)	<0.0001
γ-GTP (U/L)	54 (13-163)	51.5 (12-104)	0.31
TG (mg/dL)	246 (77-619)	131 (51-365)	<0.0001
non-HDL-C (mg/dL)	140.5 (115-165)	122.5 (105-148)	<0.0001
eGFR (mL/minute/1.73 m^2^)	71.5 (54-85)	64.5 (50-88)	0.164
UACR (mg/g)	35 (3-112)	24.5 (2-110)	0.01
hsCRP (mg/L)	3 ± 0.85	2.24 ± 0.97	<0.0001
Fatty liver index	66.8 ± 16.9	47.7 ± 20.4	<0.0001
FIB-4 index	1.30 ± 0.54	0.96 ± 0.44	<0.0001

Significant reductions were observed in all parameters except γ-GTP levels and eGFR after the switch. The median weight decreased by 4 kg, and the mean HbA1c declined by 0.89%. Additionally, decreases in the fatty liver index, FIB-4 index, UACR, and hsCRP were noted six months after switching to tirzepatide, indicating its potential to suppress MASLD, diabetic nephropathy, and chronic inflammation.

Correlation analysis of changes in parameters and changes in fatty liver index, FIB-4 index, and UACR before and after switching to tirzepatide

To explore factors associated with improvements in fatty liver disease, hepatic fibrosis, and diabetic nephropathy following the switch to tirzepatide, a correlation analysis was conducted using the changes in the fatty liver index, FIB-4 index, and UACR before and after the switch to tirzepatide (Table 3).

**Table 3:** Correlation analysis of changes in parameters and changes in the fatty liver index, FIB-4 index, and UACR before and after switching to tirzepatide The change (Δ) in each parameter was calculated by subtracting the value before the switch from the value six months after the switch. The body weight loss rate was calculated by subtracting the weight six months after the switch from the weight before the switch, dividing the result by the weight before the switch, and multiplying by 100. Pearson’s correlation coefficient was employed for correlation analyses. The values of items marked with an asterisk were log-transformed. In the table, the numbers on the left indicate R (Pearson correlation coefficient) and the numbers on the right indicate p values HbA1c, hemoglobin A1c; AST, aspartate aminotransferase; ALT, alanine aminotransferase; γ-GTP, γ-glutamyl transpeptidase; TG, triglyceride; non-HDL-C, non-high-density lipoprotein cholesterol; eGFR, estimated glomerular filtration rate; UACR, urine albumin-to-creatinine ratio; hsCRP, high-sensitive C-reactive protein; FIB-4, fibrosis 4; N/A, not applicable

Parameters	ΔFatty liver index	ΔFIB-4 index	ΔUACR
ΔBody weight (kg)^*^	0.29, 0.0479	0.162, 0.276	0.367, 0.0354
Weight loss rate (%)	-0.411, 0.002	-0.2, 0.146	-0.342, 0.0353
ΔBody mass index (kg/m^2^)	0.374, 0.005	0.218, 0.112	0.412, 0.0102
ΔWaist circumference (cm)^*^	0.328, 0.0178	0.173, 0.221	0.193, 0.252
ΔHbA1c (%)	0.379, 0.005	0.124, 0.373	0.121, 0.468
ΔAST (U/L)^*^	0.016, 0.926	0.140, 0.401	0.116, 0.563
ΔALT (U/L)^*^	0.248, 0.104	0.003, 0.983	-0.071, 0.695
Δγ-GTP (U/L)^*^	0.167, 0.362	0.160, 0.381	0.080, 0.711
ΔTG (mg/dL)^*^	0.731, <0.0001	-0.0251, 0.87	0.16, 0.365
Δnon-HDL-C (mg/dL)	-0.0159, 0.909	0.253, 0.065	-0.0272, 0.92
ΔeGFR (mL/minute/1.73 m^2^)	-0.103, 0.459	-0.0131, 0.925	0.156, 0.35
ΔUACR (mg/g)^*^	0.221, 0.182	0.274, 0.0964	N/A
ΔhsCRP (mg/L)	0.555, <0.0001	0.382, 0.004	0.511, 0.001
ΔFatty liver index	N/A	0.231, 0.0926	0.221, 0.182
ΔFIB-4 index	0.231, 0.0926	N/A	0.274, 0.0964

The changes in the fatty liver index were significantly positively correlated with changes in weight, BMI, waist circumference, HbA1c, TG, and hsCRP. Similarly, changes in the FIB-4 index exhibited a significant positive correlation with changes in hsCRP levels. For the UACR, significant positive correlations were observed with changes in weight, BMI, and hsCRP levels.

Factors predicting weight loss rate after switching to tirzepatide

Tirzepatide has been shown to improve various obesity-related metabolic disorders, primarily through weight reduction [16,18,19]. We conducted a series of analyses to evaluate whether the weight loss rate achieved by switching to tirzepatide could be predicted from clinical parameters before the switch. First, a simple regression analysis was performed, with the weight loss rate six months after switching to tirzepatide as the dependent variable and clinical findings before the switch as explanatory variables (Table 4).

**Table 4:** Association of weight loss rate after switching to tirzepatide with each explanatory factor in patients by univariate regression analysis The weight loss rate was calculated by subtracting the weight six months after the switch from the weight before the switch, dividing the result by the weight before the switch, and multiplying by 100. Independent variables are those before the switch β, partial regression coefficient; CI, confidence interval; T2DM, type 2 diabetes mellitus; HbA1c, hemoglobin A1c; AST, aspartate aminotransferase; ALT, alanine aminotransferase; γ-GTP, γ-glutamyl transpeptidase; TG, triglyceride; non-HDL-C, non-high-density lipoprotein cholesterol; eGFR, estimated glomerular filtration rate; UACR, urine albumin-to-creatinine ratio; hsCRP, high-sensitive C-reactive protein; FIB-4, fibrosis 4; SGLT2i, SGLT2, sodium glucose co-transporter 2 inhibitor; GLP-1RA, glucagon-like peptide-1 receptor agonist

Independent variables	β	95% CI	p value
Age (years)	-0.144	-0.207 to -0.081	<0.0001
Sex (0: males, 1: females)	-1.312	-2.832 to 0.207	0.089
Body mass index (kg/m^2^)	0.269	0.104 to 0.434	0.002
Duration of T2DM (years)	-0.134	-0.223 to -0.046	0.004
HbA1c (%)	-2.086	-2.646 to -1.526	<0.0001
AST (U/L)	0.029	-0.006 to 0.063	0.102
ALT (U/L)	0.026	-0.011 to 0.063	0.162
γ-GTP (U/L)	-0.025	-0.053 to 0.003	0.078
TG (mg/dL)	0.001	-0.007 to 0.009	0.815
non-HDL-C (mg/dL)	0.026	-0.014 to 0.066	0.195
eGFR (mL/minute/1.73 m^2^)	-0.036	-0.110 to 0.037	0.324
UACR (mg/g)	0.010	-0.014 to 0.034	0.414
hsCRP (mg/L)	0.183	-0.736 to 1.102	0.691
Fatty liver index	0.047	0.002 to 0.092	0.040
FIB-4 index	1.034	-0.385 to 2.453	0.150
SGLT2i (0: no, 1: yes)	0.388	-1.207 to 1.982	0.628
GLP-1RA (0: dulaglutide, 1: semaglutide)	-1.461	-2.975 to 0.054	0.058
Metformin (0: no, 1: yes)	0.884	-1.423 to 3.191	0.446
Insulin (0: no, 1: yes)	0.032	-2.162 to 2.226	0.977
Statin (0: no, 1: yes)	-0.072	-1.658 to 1.514	0.928
Fibrate (0: no, 1: yes)	-0.193	-1.824 to 1.438	0.813

Significant factors identified in this analysis included age, BMI, duration of type 2 diabetes, HbA1c level, and fatty liver index before the switch. Subsequently, a multiple regression analysis was conducted using the weight loss rate as the dependent variable and the five significant factors identified in the simple regression analysis as explanatory variables (Table 5).

**Table 5:** Association of weight loss rate after switching to tirzepatide with each explanatory factor in patients by multivariate regression analysis The weight loss rate was calculated by subtracting the weight six months after the switch from the weight before the switch, dividing the result by the weight before the switch, and multiplying by 100. Independent variables are those before the switch. All independent variables are entered simultaneously into the model ^*^p, p in the model adjusted for gender β, partial regression coefficient; CI, confidence interval; T2DM, type 2 diabetes mellitus; HbA1c, hemoglobin A1c

Independent variables	β	95% CI	p	^*^p
Age (years)	-0.060	-0.112 to -0.009	0.023	0.025
Body mass index (kg/m^2^)	0.138	-0.013 to 0.289	0.071	0.074
Duration of T2DM (years)	-0.066	-0.128 to -0.003	0.040	0.049
HbA1c (%)	-1.769	-2.310 to -1.229	<0.0001	<0.0001
Fatty liver index	-0.027	-0.065 to 0.012	0.169	0.192

This analysis revealed that age, duration of type 2 diabetes, and HbA1c level before the switch were independent predictors of weight loss rate. The significant difference among these three items persisted even in the model adjusted for gender.

Factors predicting a decline in the fatty liver index after switching to tirzepatide

We also explored whether the reduction in the fatty liver index after switching to tirzepatide could be predicted based on clinical parameters before the switch. Initially, a simple regression analysis was conducted, with the decline in the fatty liver index six months after the switch as the dependent variable and the clinical findings before the switch as explanatory variables (Table 6).

**Table 6:** Association of a decline in the fatty liver index after switching to tirzepatide with each explanatory factor in patients by univariate regression analysis The decline in the fatty liver index was determined by subtracting the fatty liver index six months after the switch from the fatty liver index before the switch. Independent variables are those before the switch β, partial regression coefficient; CI, confidence interval; T2DM, type 2 diabetes mellitus; HbA1c, hemoglobin A1c; AST, aspartate aminotransferase; ALT, alanine aminotransferase; γ-GTP, γ-glutamyl transpeptidase; TG, triglyceride; non-HDL-C, non-high-density lipoprotein cholesterol; eGFR, estimated glomerular filtration rate; UACR, urine albumin-to-creatinine ratio; hsCRP, high-sensitive C-reactive protein; FIB-4, fibrosis 4; SGLT2i, SGLT2, sodium glucose co-transporter 2 inhibitor; GLP-1RA, glucagon-like peptide-1 receptor agonist

Independent variables	β	95% CI	p
Age (years)	-0.417	-0.735 to -0.099	0.011
Sex (0: males, 1: females)	-2.052	-9.082 to 4.978	0.561
Body mass index (kg/m^2^)	0.013	-0.805 to 0.831	0.975
Duration of T2DM (years)	-0.494	-0.906 to -0.081	0.020
HbA1c (%)	-2.340	-5.923 to 1.242	0.196
AST (U/L)	-0.028	-0.188 to 0.131	0.723
ALT (U/L)	-0.061	-0.230 to 0.108	0.474
γ-GTP (U/L)	-0.113	-0.240 to 0.015	0.081
TG (mg/dL)	0.0130	-0.023 to 0.049	0.474
non-HDL-C (mg/dL)	0.010	-0.173 to 0.194	0.909
eGFR (mL/minute/1.73 m^2^)	-0.070	-0.404 to 0.264	0.676
UACR (mg/g)	0.021	-0.088 to 0.130	0.705
hsCRP (mg/L)	-2.377	-6.477 to 1.723	0.250
Fatty liver index	0.056	-0.154 to 0.266	0.596
FIB-4 index	3.787	-2.662 to 10.236	0.244
SGLT2i (0: no, 1: yes)	-2.502	-9.683 to 4.678	0.488
GLP-1RA (0: dulaglutide, 1: semaglutide)	1.130	-5.941 to 8.201	0.750
Metformin (0: no, 1: yes)	3.437	-6.990 to 13.864	0.511
Insulin (0: no, 1: yes)	-7.340	-17.027 to 2.347	0.134
Statin (0: no, 1: yes)	2.565	-4.557 to 9.688	0.473
Fibrate (0: no, 1: yes)	-1.318	-8.675 to 6.038	0.721

This analysis identified age and duration of type 2 diabetes before the switch as significant factors. Subsequently, a multiple regression analysis was performed using the decline in the fatty liver index as the dependent variable and the two significant factors from the simple regression analysis as explanatory variables (Table 7).

**Table 7:** Association of a decline in the fatty liver index after switching to tirzepatide with each explanatory factor in patients by multivariate regression analysis The decline in the fatty liver index was determined by subtracting the fatty liver index six months after the switch from the fatty liver index before the switch. Independent variables are those before the switch. All independent variables are entered simultaneously into the model ^*^p, p in the model adjusted for gender β, partial regression coefficient; CI, confidence interval; T2DM, type 2 diabetes mellitus

Independent variables	β	95% CI	p	^*^p
Age (years)	-0.318	-0.656 to 0.020	0.064	0.066
Duration of T2DM (years)	-0.342	-0.776 to 0.092	0.120	0.125

While neither factor reached statistical significance in the multiple regression model, age before the switch showed a trend toward significance as a potential predictor of fatty liver index reduction (p = 0.064). In addition, even in the model adjusted for gender, the p values for age were similar (p = 0.066).

Relationship between appetite suppression after switching to tirzepatide and changes in parameters

Tirzepatide enhances the action of GIP, which has appetite-suppressing effects, in addition to the appetite suppression seen with conventional GLP-1RAs [29], suggesting that the extent of appetite suppression significantly influences its efficacy. To evaluate this, we compared the changes in various parameters based on whether appetite suppression occurred after switching from GLP-1RAs to tirzepatide (Table 8).

**Table 8:** Relationship between appetite suppression after switching to tirzepatide and parameter changes Data are expressed as the mean ± standard deviation, the median (range), or number (%), and are compared using Student’s t test, the Mann-Whitney U test, or Fisher's exact test. Logistic regression analysis was used for comparative analysis, adjusted for age. The change (Δ) in each parameter was calculated by subtracting the value before the switch from the value six months after the switch. The weight loss rate was calculated by subtracting the weight six months after the switch from the weight before the switch, dividing the result by the weight before the switch, and multiplying by 100 ^*^p, p in the model adjusted for age HbA1c, hemoglobin A1c; AST, aspartate aminotransferase; ALT, alanine aminotransferase; γ-GTP, γ-glutamyl transpeptidase; TG, triglyceride; non-HDL-C, non-high-density lipoprotein cholesterol; eGFR, estimated glomerular filtration rate; UACR, urine albumin-to-creatinine ratio; hsCRP, high-sensitive C-reactive protein; FIB-4, fibrosis 4; SGLT2i, SGLT2, sodium glucose co-transporter 2 inhibitor; GLP-1RA, glucagon-like peptide-1 receptor agonist, N/A, not available

Parameters	Yes (n = 35)	No (n = 19)	p	^*^p
Females, n (%)	14 (40)	11 (58)	0.259	0.302
Age (years)	54.5 ± 10.4	62.3 ± 8.76	0.007	N/A
ΔBody weight (kg)	-2.9 (-9.2 to 1.7)	-1 (-4.9 to 0.2)	0.001	0.037
Weight loss rate (%)	4.05 ± 2.97	1.76 ± 1.81	0.003	0.071
ΔBody mass index (kg/m^2^)	-1.22 ± 0.93	-0.45 ± 0.47	0.002	0.036
ΔWaist circumference (cm)	-5 (-14 to 2)	-4 (-6 to 0)	0.008	0.029
ΔHbA1c (%)	-1.23 ± 0.88	-0.25 ± 0.87	0.0002	0.012
ΔAST (U/L)	-6 (-148 to 9)	-2 (-15 to 13)	0.063	0.090
ΔALT (U/L)	-9 (-126 to 3)	-4 (-21 to 12)	0.001	0.009
Δγ-GTP (U/L)	-5 (-59 to 19)	2 (-21 to 30)	0.018	0.027
ΔTG (mg/dL)	-108 (-318 to 14)	-26 (-150 to 34)	0.001	0.008
Δnon-HDL-C (mg/dL)	-15.5 ± 21.8	-16.2 ± 14.1	0.908	0.818
ΔeGFR (mL/minute/1.73 m^2^)	-3.63 ± 17.2	-2.32 ± 14.8	0.78	0.772
ΔUACR (mg/g)	-12 (-91 to 91)	7 (-24 to 44)	0.0002	0.036
ΔhsCRP (mg/L)	-1.29 ± 0.66	0.20 ± 0.68	<0.0001	0.001
ΔFatty liver index	-23.8 ± 11.3	-10.6 ± 11.0	0.0001	0.004
ΔFIB-4 index	-0.42 ± 0.52	-0.19 ± 0.50	0.126	0.424
SGLT2i, n (%)	24 (69)	9 (47)	0.153	0.092
GLP-1RA before tirzepatide initiation, n (%)				
Dulaglutide	20 (57)	4 (21)	0.021	0.048
Semaglutide	15 (43)	15 (79)	-	-
Metformin	32 (91)	15 (79)	0.226	0.209
Insulin	3 (9)	5 (26)	0.113	0.188
Statin	20 (57)	12 (34)	0.775	0.620
Fibrate	23 (66)	12 (34)	1	0.899

In the group that experienced appetite suppression, the reductions in parameters, excluding AST, non-HDL-C, eGFR, and the FIB-4 index, were significantly greater. Furthermore, this group had a higher proportion of patients who had been using dulaglutide as their prior GLP-1RA and was significantly younger in age. Importantly, even after adjusting for age, the significant differences in parameter changes between the two groups persisted.

Relationship between co-use of SGLT2is and parameter changes

The effectiveness of SGLT2is in patients with MASLD has been widely documented [10,12-14]. Moreover, their use is recommended in the guidelines for MASLD both in Japan and other countries [4,30]. Consequently, the combined use of tirzepatide and SGLT2is is likely to increase in clinical practice. To evaluate the potential differences in the therapeutic effects of switching to tirzepatide based on SGLT2i usage, we analyzed the changes in various parameters between the SGLT2i combination and noncombination groups (Table 9).

**Table 9:** Relationship between co-use of SGLT2is and parameter changes Data are expressed as the mean ± standard deviation, the median (range), or number (%), and are compared using Student’s t test, the Mann-Whitney U test, or Fisher's exact test. The change (Δ) in each parameter was calculated by subtracting the value before the switch from the value six months after the switch. The weight loss rate was calculated by subtracting the weight six months after the switch from the weight before the switch, dividing the result by the weight before the switch, and multiplying by 100 HbA1c, hemoglobin A1c; AST, aspartate aminotransferase; ALT, alanine aminotransferase; γ-GTP, γ-glutamyl transpeptidase; TG, triglyceride; non-HDL-C, non-high-density lipoprotein cholesterol; eGFR, estimated glomerular filtration rate; UACR, urine albumin-to-creatinine ratio; hsCRP, high-sensitive C-reactive protein; FIB-4, fibrosis 4; SGLT2i, SGLT2, sodium glucose co-transporter 2 inhibitor

Parameters	Yes (n = 33)	No (n = 21)	p
Females, n (%)	14 (42)	11 (52)	0.579
Age (years)	58.5 ± 10.2	55.2 ± 10.8	0.271
Appetite suppression yes, n (%)	24 (73)	11 (52)	0.153
ΔBody weight (kg)	-2.7 (-9.1 to 1.7)	-1.2 (-9.2 to 0.2)	0.295
Weight loss rate (%)	3.39 ± 2.72	3.01 ± 3.03	0.628
ΔBody mass index (kg/m^2^)	-0.98 ± 0.82	-0.90 ± 0.98	0.748
ΔWaist circumference (cm)	-5 (-14 to 2)	-3 (-10 to 0)	0.030
ΔHbA1c (%)	-0.97 ± 1.02	-0.76 ± 0.94	0.452
ΔAST (U/L)	-5 (-148 to 13)	-1 (-11 to 7)	0.071
ΔALT (U/L)	-8 (-126 to 11)	-6 (-22 to 12)	0.307
Δγ-GTP (U/L)	-3 (-59 to 29)	-2 (-36 to 30)	0.505
ΔTG (mg/dL)	-75 (-318 to 23)	-91 (-283 to 34)	0.818
Δnon-HDL-C (mg/dL)	-13.7 ± 19.1	-19.0 ± 19.7	0.335
ΔeGFR (mL/minute/1.73 m^2^)	-4.33 ± 17.3	-1.33 ± 14.7	0.514
ΔUACR (mg/g)	-8 (-82 to 44)	-3 (-91 to 91)	0.972
ΔhsCRP (mg/L)	-0.80 ± 0.87	-0.70 ± 1.14	0.729
ΔFatty liver index	-18.2 ± 11.4	-20.7 ± 14.8	0.488
ΔFIB-4 index	-0.29 ± 0.51	-0.42 ± 0.54	0.351

Among the parameters assessed, a significantly greater reduction in waist circumference was observed in the SGLT2i combination group, while no significant differences were noted for other items.

Relationship between the type of GLP-1RA used before switching to tirzepatide and parameter changes

Tirzepatide, an incretin-related drug like GLP-1RAs, may increasingly replace existing GLP-1RAs depending on future large-scale clinical trial outcomes demonstrating its organ-protective effects. To assess whether the therapeutic effects of tirzepatide vary based on the type of GLP-1RA previously used, we analyzed the clinical outcomes according to the prior use of semaglutide or dulaglutide (Table 10).

**Table 10:** Relationship between the type of GLP-1RA used before switching to tirzepatide and parameter changes Data are expressed as the mean ± standard deviation, the median (range), or number (%), and are compared using Student’s t test, the Mann-Whitney U test, or Fisher's exact test. Logistic regression analysis was used for comparative analysis adjusted for age. The change (Δ) in each parameter was calculated by subtracting the value before the switch from the value six months after the switch. The weight loss rate was calculated by subtracting the weight six months after the switch from the weight before the switch, dividing the result by the weight before the switch, and multiplying by 100 ^*^p, p in the model adjusted for age HbA1c, hemoglobin A1c; AST, aspartate aminotransferase; ALT, alanine aminotransferase; γ-GTP, γ-glutamyl transpeptidase; TG, triglyceride; non-HDL-C, non-high-density lipoprotein cholesterol; eGFR, estimated glomerular filtration rate; UACR, urine albumin-to-creatinine ratio; hsCRP, high-sensitive C-reactive protein; FIB-4, fibrosis 4; GLP-1RA, glucagon-like peptide-1 receptor agonist; N/A, not available

Parameters	Dulaglutide (n = 24)	Semaglutide (n = 30)	p	^*^p
Females, n (%)	8 (33)	17 (57)	0.106	0.145
Age (years)	53.9 ± 11.0	59.9 ± 9.39	0.037	N/A
Appetite suppression yes, n (%)	20 (83)	15 (50)	0.021	0.048
ΔBody weight (kg)	-3.15 (-9.2 to 1.7)	-1.2 (-8.5 to 0.2)	0.024	0.259
Weight loss rate (%)	4.05 ± 3.13	2.59 ± 2.41	0.058	0.340
ΔBody mass index (kg/m^2^)	-1.23 ± 0.98	-0.72 ± 0.72	0.031	0.223
ΔWaist circumference (cm)	-5 (-14 to 2)	-4 (-10 to 0)	0.050	0.115
ΔHbA1c (%)	-1.26 ± 1.00	-0.58 ± 0.89	0.011	0.061
ΔAST (U/L)	-5 (-148 to 7)	-2.5 (-19 to 13)	0.28	0.282
ΔALT (U/L)	-8.5 (-126 to 7)	-8 (-28 to 12)	0.236	0.358
Δγ-GTP (U/L)	-5.5 (-59 to 29)	1 (-36 to 30)	0.219	0.515
ΔTG (mg/dL)	-75 (-318 to 24)	-79.5 (-279 to 34)	0.958	0.528
Δnon-HDL-C (mg/dL)	-11.3 ± 21.6	-19.3 ± 16.8	0.128	0.108
ΔeGFR (mL/minute/1.73 m^2^)	-3.42 ± 18.6	-2.97 ± 14.4	0.921	0.786
ΔUACR (mg/g)	-10.5 (-91 to 91)	-3.5 (-82 to 44)	0.288	0.847
ΔhsCRP (mg/L)	-1.04 ± 0.79	-0.54 ± 1.06	0.059	0.233
ΔFatty liver index	-18.5 ± 12.2	-19.6 ± 13.3	0.75	0.251
ΔFIB-4 index	-0.29 ± 0.36	-0.38 ± 0.62	0.554	0.200

The group that had used semaglutide before switching to tirzepatide showed significantly fewer cases of appetite suppression postswitch compared with the dulaglutide group. Additionally, the semaglutide group exhibited smaller reductions in weight, BMI, and HbA1c levels. However, there were no significant differences between the groups regarding the reduction in MASLD markers such as the fatty liver index and FIB-4 index. Notably, a significant age difference was observed between the groups. After adjusting for age, the significant differences between the groups disappeared, except for the outcome of appetite suppression.

Changes in parameters before and after switching to tirzepatide in the group using semaglutide before the switch

The prior analysis indicated a comparatively lower therapeutic effect of tirzepatide in patients previously using semaglutide. Given the widespread use of semaglutide alongside dulaglutide among GLP-1RAs in Japan, we assessed whether switching to tirzepatide provided measurable benefits within this subgroup (Table 11).

**Table 11:** Changes in parameters before and after switching to tirzepatide in the group using semaglutide before the switch (n = 30) Data are expressed as the mean ± standard deviation or the median (range), and are compared using the paired t-test or the Wilcoxon signed-rank sum test HbA1c, hemoglobin A1c; AST, aspartate aminotransferase; ALT, alanine aminotransferase; γ-GTP, γ-glutamyl transpeptidase; TG, triglyceride; non-HDL-C, non-high-density lipoprotein cholesterol; eGFR, estimated glomerular filtration rate; UACR, urine albumin-to-creatinine ratio; hsCRP, high-sensitive C-reactive protein; FIB-4, fibrosis 4

Parameters	Before the switch	Six months after the switch	p
Body weight (kg)	69.8 (56.1-95.8)	66.9 (55-93.7)	<0.0001
Body mass index (kg/m^2^)	26.7 (23.1-31.5)	25.8 (22.5-30.9)	<0.0001
Waist circumference (cm)	88 (74-101)	84 (72-99)	<0.0001
HbA1c (%)	8.11 ± 1.63	7.55 ± 1.71	0.001
AST (U/L)	28 (11-46)	25 (11-40)	0.026
ALT (U/L)	38.5 (13-55)	30 (15-51)	0.002
γ-GTP (U/L)	54 (13-97)	52.5 (13-98)	1
TG (mg/dL)	250 (77-369)	117.5 (51-354)	<0.0001
non-HDL-C (mg/dL)	144 (118-165)	122 (105-145)	<0.0001
eGFR (mL/minute/1.73 m^2^)	70 (54-85)	64 (52-86)	0.206
UACR (mg/g)	35 (5-103)	22.5 (3-109)	0.147
hsCRP (mg/L)	2.71 ± 0.82	2.19 ± 1.10	0.009
Fatty liver index	63.1 ± 19.0	44.1 ± 18.9	<0.0001
FIB-4 index	1.33 ± 0.56	0.96 ± 0.41	0.002

Our results demonstrated significant reductions across most parameters, excluding γ-GTP, eGFR, and UACR, six months after switching to tirzepatide. Notably, decreases in the fatty liver index and FIB-4 index highlighted improvements in liver-related outcomes, confirming that switching to tirzepatide yields therapeutic benefits even in patients transitioning from semaglutide.

## Discussion

In this study, we conducted a retrospective analysis to assess the six-month therapeutic effects of switching from GLP-1RAs to tirzepatide in patients with type 2 diabetes and MASLD. Our results demonstrated a reduction in body weight and HbA1c levels, along with improvements in markers of MASLD and diabetic nephropathy in this population. The multiple regression analysis identified age, duration of type 2 diabetes, and HbA1c level before switching to tirzepatide as significant predictors of weight loss rate. Additionally, age before the switch may serve as a useful predictor of a decrease in the fatty liver index. Our study also revealed that the appetite-suppressing effect of tirzepatide varied depending on the type of GLP-1RA used prior to the switch. The tirzepatide treatment was effective even in the group that had been using semaglutide.

In this study, the decrease in the fatty liver index after switching to tirzepatide was associated with improvements in obesity and hypertriglyceridemia, suggesting that obesity-induced fatty liver disease improved with weight loss. These findings align with previous studies that have reported tirzepatide's effectiveness in improving MASLD biomarkers in patients with type 2 diabetes [20,21]. Furthermore, the SYNERGY-NASH study, which evaluated the efficacy and safety of tirzepatide in patients with MASH and hepatic fibrosis confirmed by liver biopsy, reported that the tirzepatide group significantly outperformed the placebo group in terms of MASH resolution, the primary endpoint, at 52 weeks [31]. In this previously reported study, the proportion of participants meeting the criteria for MASH resolution was 10% in the placebo group, 44% in the tirzepatide 5 mg group, 56% in the tirzepatide 10 mg group, and 62% in the tirzepatide 15 mg group. These results demonstrate that approximately half of the participants in each dose group achieved resolution of MASH and that tirzepatide has a strong inhibitory effect on MASLD. This study also showed improvements in liver fibrosis, nonalcoholic fatty liver disease activity score, and its individual components, such as steatosis, lobular inflammation, and hepatocyte ballooning [31]. In addition to weight loss, other mechanisms may contribute to tirzepatide's impact on liver pathology. The activation of GIP receptors in subcutaneous white adipose tissue has been shown to improve insulin sensitivity through increased postprandial TG uptake [32,33]. Moreover, animal model studies have demonstrated that GIP receptor agonists can improve insulin resistance independently of body weight changes [34]. Furthermore, clinical trials have demonstrated that tirzepatide improves insulin sensitivity more effectively than existing GLP-1RAs [35,36]. In animal models, improving insulin sensitivity in white adipose tissue has been shown to reduce ectopic fat deposition in the liver [37]. Therefore, it is possible that tirzepatide's direct protective effect on various tissues, including adipose tissue, plays a role in improving the pathology of MASLD. Further basic research is needed to confirm these mechanisms. In this study, the decrease in the UACR after switching to tirzepatide correlated with the decrease in body weight and BMI, suggesting that it may be influenced by weight loss. Indeed, it has been reported that improvement of obesity helps suppress renal events in patients with obesity and type 2 diabetes [38]. Recently, a post hoc analysis of the SURPASS-4 study compared eGFR trends in two groups of patients with type 2 diabetes and high cardiovascular risk: a tirzepatide group and an insulin glargine group. The results showed that tirzepatide slowed the rate of decline in eGFR [39]. Interestingly, the study also found that the change in eGFR did not correlate with the change in body weight, suggesting the possibility of a weight-independent pathway for renal protection. In fact, many basic mechanisms for the renal protective effect of tirzepatide have been reported [40]; however, the existence of a direct renal protective effect of tirzepatide requires further investigation through large-scale clinical trials.

The decrease in hsCRP levels after the switch in this study suggests that tripeptide has an anti-inflammatory effect, which aligns with previous reports [41]. As previous studies have shown that tripeptide reduces various cardiovascular risk markers such as hsCRP and leptin [41], it may be pertinent to investigate the cardiovascular event suppression effects among the participants in this study in the future.

Our multiple regression analysis indicated that young age, short duration of type 2 diabetes, and low HbA1c level before switching to tirzepatide were predictive factors for weight loss after the switch, and that young age before the switch may be a predictor for a decrease in the fatty liver index. Previous reports have identified high doses of tirzepatide, female sex, Caucasian/Asian race, young age, metformin use, good glycemic control, and low non-HDL-C levels as predictors of weight loss with tirzepatide use [42]. Additionally, low HbA1c levels and a short duration of diabetes have been identified as predictors of the blood glucose-lowering effect of tirzepatide [43]. Some overlap exists between the predictive factors in these previous reports and the findings in our study; hence, further research into predictors of MASLD improvement with tirzepatide, including prospective studies, may be necessary.

In this study, tirzepatide suppressed appetite in more than half of the patients. The decrease in the fatty liver index was significantly greater in the group that experienced appetite suppression, suggesting that the presence or absence of appetite suppression could be an indicator of tirzepatide's therapeutic effect. Additionally, the group that experienced appetite suppression was significantly more likely to have been using dulaglutide before the switch. Previous studies comparing the therapeutic effects of dulaglutide and semaglutide have shown that the dulaglutide group exhibited less weight loss and blood glucose suppression effects [44,45]. Therefore, it is possible that appetite suppression became relatively stronger after switching to tirzepatide among dulaglutide users due to weaker appetite suppression before the switch.

Our results also indicated that there was little difference in the effectiveness of switching to tirzepatide based on whether or not an SGLT2i was used in combination. As previously mentioned, SGLT2is are drugs that lower blood glucose levels independently of insulin by increasing urinary glucose excretion [46]. They are highly recommended in the guidelines for MASLD in Japan and other countries [4,30]. Moreover, the use of SGLT2is in combination with tirzepatide is expected to increase in the future. Therefore, our finding that the concomitant use of an SGLT2i does not suppress the therapeutic effects of tirzepatide may be significant.

We found that the appetite suppression effect was stronger in the group that had used dulaglutide before switching to tirzepatide. In our study, semaglutide, a GLP-1RA with a relatively strong appetite-suppressing effect, was used at the highest dose in Japan (1 mg/week) in the semaglutide group, which may have limited the effect of switching to tirzepatide. However, after adjusting for age, the significant differences between the two groups for each parameter, other than appetite suppression, disappeared, suggesting that the type of GLP-1RA used before switching had little impact on the therapeutic outcomes. Furthermore, in the semaglutide group, there was a significant decrease in weight, BMI, HbA1c level, fatty liver index, and FIB-4 index 6 months after switching to tirzepatide, indicating that even high-dose semaglutide users may benefit from switching to tirzepatide.

When introducing a new drug, the primary concern is adverse effects. In the above-mentioned prospective study on tirzepatide and MASLD, the most common adverse events observed in the tirzepatide group during the 52-week intervention period were gastrointestinal disorders, with the majority being mild or moderate in severity, and no patients experienced severe adverse effects. There is currently no data on the safety of very long-term use, and this will likely be clarified in future prospective studies.

This study had a few limitations. First, the majority of cases were switched to tirzepatide during a period when long-term prescriptions were not available, resulting in a high frequency of hospital visits and a dosage limit of 5 mg/week due to limited shipments. Therefore, in the future, it may be necessary to evaluate cases where tirzepatide was introduced following the lifting of long-term prescription restrictions. Second, the small number of subjects in this study prevented the simultaneous entry of all items previously reported as associated factors for effects of tirzepatide, such as metformin background therapy and non-HDL-C, into the multivariate analysis, potentially weakening the statistical power of the findings. Third, regarding the evaluation of fatty liver disease and liver fibrosis, this study did not involve interventions by a hepatologist, and liver biopsy was not performed. Fatty liver was diagnosed by imaging tests before switching to tirzepatide; however, due to the retrospective nature of this study, there were few cases where imaging tests were performed six months after the switch. Therefore, two markers, the fatty liver index and FIB-4 index, were used to evaluate changes before and after the switch. Fourth, this study did not include a control group that did not use tirzepatide. In Japan, prospective, long-term randomized controlled trials are eagerly awaited to investigate the effects of tirzepatide on MASLD. Also, future prospective studies in Japan are needed to provide insights into whether tirzepatide use influences the progression of liver pathology in patients with MASLD, particularly those that track changes in liver biopsy or imaging findings over the long term.

## Conclusions

We conducted a retrospective analysis to assess the six-month therapeutic effects of switching from GLP-1RAs to tirzepatide in patients with type 2 diabetes and MASLD. This study suggested the efficacy of switching from GLP-1RAs to tirzepatide among Japanese patients with type 2 diabetes and MASLD. The multiple regression analysis identified age, duration of type 2 diabetes, and HbA1c level before switching to tirzepatide as significant predictors of weight loss rate. Additionally, we found that the therapeutic effect of tirzepatide varied depending on the type of GLP-1RA used prior to the switch and it can be expected even in patients who were using semaglutide before the switch. Tirzepatide demonstrates great potential for the management of MASLD by promoting weight loss, improving glycemic control, and reducing chronic inflammation compared to GLP-1RAs.
